# The Interaction between Amyloid-*β* Peptides and Anionic Lipid Membranes Containing Cholesterol and Melatonin

**DOI:** 10.1371/journal.pone.0099124

**Published:** 2014-06-10

**Authors:** Hannah Dies, Laura Toppozini, Maikel C. Rheinstädter

**Affiliations:** Department of Physics and Astronomy, McMaster University, Hamilton, Ontario, Canada; University of Waterloo, Canada

## Abstract

One of the hallmarks of Alzheimer's disease is the formation of senile plaques, primarily consisting of amyloid-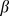 (A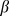) peptides. Peptide-membrane and peptide-lipid interactions are thought to be crucial in this process. We studied the interaction of A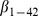 and A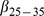 peptides with anionic lipid membranes made of dimyristoylphosphatidylcholine (DMPC) and dimyristoylphosphoserine (DMPS) using X-ray diffraction. We compare the experimentally determined electron densities in the gel state of the membranes with density calculations from peptide structures reported in the Protein Data Bank in order to determine the position of the peptide in the bilayers. The full length peptide A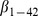 was found to embed in the hydrocarbon core of the anionic lipid bilayers. Two populations were found for the A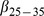 peptide: (1) membrane-bound states in the hydrophilic head group region of the bilayers, where the peptides align parallel to the membranes, and (2) an embedded state in the bilayer center. Aging plays an important role in the development of Alzheimer's, in particular with respect to changes in cholesterol and melatonin levels in the brain tissue. Immiscible cholesterol plaques were created by addition of 30 mol% cholesterol to the anionic membranes. The A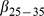 peptides were found to strongly interact with the lipid bilayers, displacing further cholesterol molecules into the plaques, effectively lowering the cholesterol concentration in the membranes and increasing the total fraction of cholesterol plaques. Addition of 30 mol% melatonin molecules to the anionic membranes drastically reduced the population of the membrane-embedded A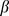 state. These results present experimental evidence for an interaction between A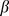 peptides, melatonin and cholesterol in lipid membranes.

## Introduction

A primary feature in the pathogenesis of Alzheimer's disease is the deposition of insoluble fibrillar plaques in the extracellular space of brain tissue. The major component of these plaques is the amyloid-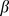 peptide (A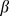). In most patients with Alzheimer's disease, symptoms first appear after age 60. While the aggregation of proteins appears to be to some extent an inherent part of aging [1], increasing evidence suggests a link between the neurodegenerative disease and changes in the composition of brain tissue. In particular, age-related changes in cholesterol [2]–[4] and melatonin [5]–[8] levels have been linked to the development of Alzheimer's disease.

The interactions of proteins and peptides with lipid membranes play a large role in maintaining the integrity and functionality of the cell membrane, and significant changes in these interactions are involved in the pathology of many diseases [9]. Misfolding and aggregation of A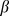 peptides, specifically, is involved in the development of Alzheimer's disease, although the exact relationship between the protein structure and the pathology of Alzheimer's is still unclear [10]. A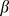 consists of a polypeptide with 42 amino acids, 10 of which (segment 25–35) comprise the transmembrane segment of the amyloid precursor protein (APP) and also comprise part of the full length A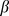 peptide. As such, this short transmembrane segment is often used in studies of the protein interactions and partitioning in the membrane [11], [12].

While A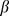 peptides are frequently reported in an extracellular location, A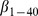 and A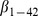 molecules were found to strongly interact with negatively charged lipids and to bind to anionic, negatively charged membranes [13]–[19], orienting parallel to the membrane surface. A small percentage of charged phospholipids is found in cell membranes in the brain and is essential for proper cell signalling, protein sorting, and cell adhesion [20]. Through X-ray and neutron diffraction, Mason *et al.* [21] and Dante, Hauβ and Dencher [11] observed an embedded state for the A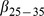 segment in anionic lipid membranes. Dante *et al.* presented subsequent evidence that the full-length A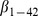 peptide is capable of penetrating lipid membranes [22]. The exact locations of the peptides for both the membrane-bound state and the embedded state are prerequisites for understanding the interactions of the peptide with the membrane. They are of particular importance as the interaction between membrane-bound peptides is a membrane-mediated elastic interaction with strong dependence on the membrane environment [23]–[25], rather than a direct peptide-peptide interaction.

Physiological concentrations of cholesterol have been shown to play a significant role in the interactions of the A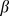 peptide with cell membranes [12], [26]. Several studies have made correlations between high cholesterol concentrations and increased risk of Alzheimer's disease [27], [28]. Cholesterol lowering drugs (statins) have been shown to reduce the risk of Alzheimer's disease [29]. Conversely, melatonin, a pineal hormone involved in regulating the circadian cycle, has been shown to have the opposite effect; it acts as an antioxidant and may also inhibit the formation of toxic amyloid structures [26]. While cholesterol leads to a decrease in fluidity, melatonin is a largely hydrophilic amino acid derivative hormone, which has been shown to reside in the head group region [30], increasing membrane fluidity and causing a corresponding increase in head group area and a decrease in bilayer thickness [30]–[32]. Increased fluidification of the membranes is speculated to inhibit peptide insertion, as A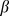 has been found to preferably interact with gel phase membranes [33]. Although cholesterol and melatonin are highly integrated into the biochemical pathways of the cell, previous studies have identified that their mechanism of influence on A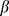 is not purely metabolic; many of their effects are as a result of biophysical interactions that influence the protein and membrane structure [31], [34].

Cholesterol has been speculated to be distributed non-randomly into domains within biological and model membranes [35]. This may result in the formation of so-called *rafts*, nanometer sized transient functional domains, which are thought to take part in membrane-associated events such as signal transduction, cell adhesion, signalling, cell trafficking and lipid/protein sorting [36]–[53]. Small, transient cholesterol domains at physiological cholesterol levels were recently reported in binary lipid (DPPC) bilayers from computer simulations [54] and experiments [53], [55]. These fluctuating domains are the precursors of immiscible cholesterol plaques at high cholesterol concentrations [56]–[65] and are speculated to have significance in early stages of atherosclerosis and the formation of of atherosclerotic plaques [63].

In this study, anionic lipid membranes were prepared with 97 mol% 1,2-dimyristol-sn-glycero-3-phosphatidylcholine (DMPC), a 14 chain saturated phospholipid with an overall zwitterionic nature, and 3 mol% 1,2-dimyristol-sn-glycero-3-phospho-L-serine (DMPS), a 14 chain saturated anionic phospholipid. The goal of this work was to study the interaction between amyloid-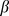 peptides and DMPC/DMPS model membranes. as DMPC is commonly used in model membrane studies. High resolution X-ray diffraction measurements from multi-lamellar membrane stacks were used to determine the location of the full length A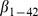 peptide and the A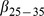 fragment. We compared the experimentally determined electron density to the calculated electron distribution of the peptides to determine their location in the bilayers. In order to permit measurement of higher order Bragg peaks, and thereby achieve a high spatial resolution, the membranes were studied in their gel state. While A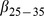 peptides were found in both membrane-bound and embedded states, the A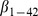 monomer was found in an embedded state in the bilayers, only.

The interactions between the A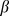 peptides, cholesterol and melatonin were studied in fluid phase bilayers, in the physiologically relevant state. Immiscible cholesterol plaques were created by addition of 30 mol% cholesterol to the anionic membranes. The addition of A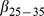 to the cholesterol-containing membranes resulted in an expulsion of cholesterol molecules from the bilayers and an increase in the total fraction of cholesterol plaques in the membrane. The presence of melatonin was found to inhibit the insertion of A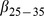 into the hydrophobic membrane core in the fluid state of the bilayers.

## Results

Eight membrane complexes were prepared for this study, as detailed in the Materials and Methods Section and listed in Table 1. The anionic lipid matrix was made of 97 mol% DMPC+3 mol% DMPS. The location of the peptides in the matrix was first studied by inclusion of 3 mol% A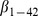 and A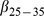. We compare the electron densities of the bilayers without peptides and in the presence of the peptides with the electron distribution of peptide structures published in the Protein Data Bank. The position of the peptides was determined by translating and rotating the molecules until good agreement between experiment and calculation was achieved. As high resolution structural data are needed for this technique, which involves the collection of high order Bragg peaks, these studies were conducted in a de-hydrated gel state of the membranes.

**Table 1:** List of all the samples prepared for this study and their molecular composition.

Sample	DMPC	DMPS	Cholesterol	Melatonin	Amyloid- 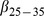	Amyloid- 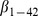	relative humidity	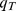	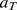	Area per lipid	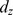
	(mol%)	(mol%)	(mol%)	(mol%)	(mol%)	(mol%)	(%)	(Å 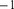 )	(Å)	(Å 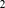 )	(Å)
1	97	3	–	–	–	–	50	1.49	4.86	40.95	55.07
2	97	3	–	–	3	–	50	1.49	4.85	40.79	52.41
3	97	3	–	–	–	3	50	1.49	4.86	41.04	55.02
4	97	3	–	—	–	–	100	1.48	4.87	60.6[79]	68.97
5	97	3	–	30	–	–	100	1.48	4.89	60.6[79]	67.52
6	97	3	–	30	3	–	100	1.48	4.89	60.6[79]	66.74
7	97	3	30	–	–	–	100	1.42	4.39	60.6[79]	65.54
8	97	3	30	–	3	–	100	1.45	4.33	60.6[79]	68.21

The anionic lipid matrix was formed by a mixture of saturated DMPC and charged DMPS lipid molecules. The full length amyloid-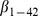 peptide and the amyloid-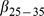 segment were added at a concentration of 3 mol%. 30 mol% cholesterol and melatonin were added (separately) to study the interactions of these molecules with A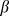 peptides. 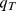 denotes the position of the acyl chain correlation peak. 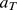 is the corresponding hydrocarbon tail spacing. 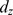 refers to the lamellar spacing, i.e., the distance between two neighboring membranes in the membrane stacks. The area per lipid for the gel state samples was determined from 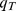. Lipid areas in the fluid state can not be determined using this technique; therefore, we used a lipid area of 60.6 Å, as reported by Kučerka *et al.* [79] for fluid DMPC membranes.

To study the interaction between peptides and cholesterol, 30 mol% cholesterol was added to the matrix to form immiscible cholesterol plaques, followed by the inclusion of A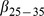. The interaction between A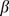 and melatonin was investigated by preparing 97 mol% DMPC/3 mol% DMPS with 30 mol% melatonin and 3 mol% of A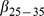. All samples in this study (Table 1) were incubated for 15 hours at 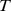 = 30°C in a 100% H
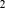
O atmosphere prior to the experiments.

**Figure 1:**
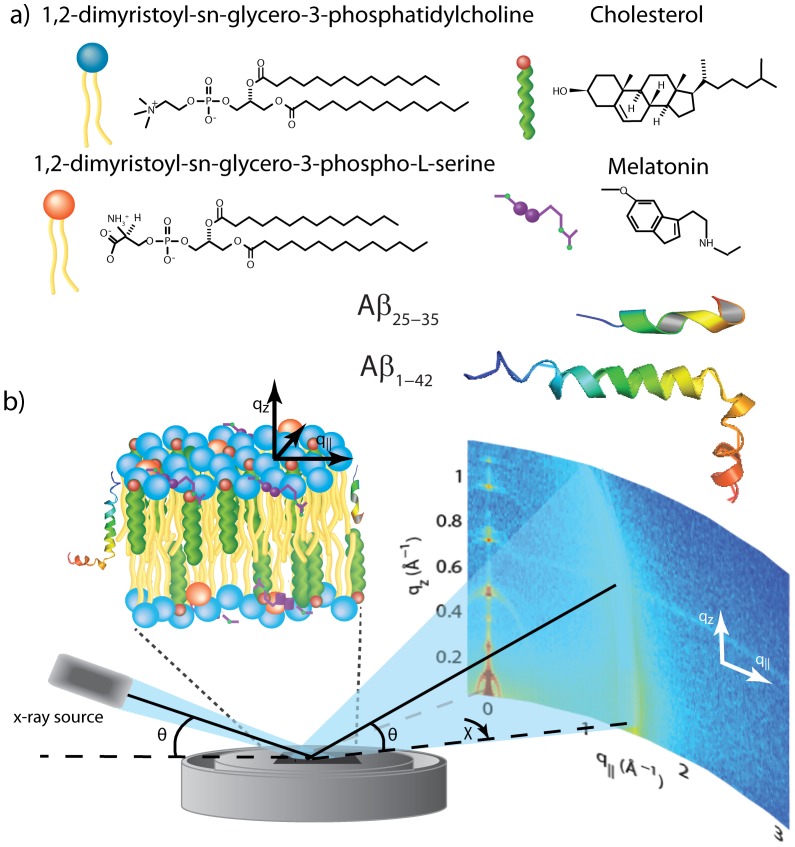
The materials and apparatus used for the experiment. (a) Schematic representations of DMPC, DMPS, cholesterol, melatonin, amyloid-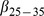 and amyloid-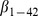 molecules. (b) Diagram of the experimental setup used for the X-ray diffraction measurements. Two-dimensional data sets were collected to study molecular structure perpendicular to the solid supported membranes (out-of-plane) and parallel to the membranes (in-plane).

As depicted in Figure 1 b), the samples were oriented such that the 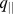 axis probed lateral membrane structure and the perpendicular axis, 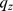, probed out-of-plane structure of the multi-lamellar membrane complexes. The samples were kept in a temperature and humidity controlled chamber (humidity chamber) during the measurements.

Fully hydrated liquid crystalline samples are generally assumed to best mimic physiologically relevant conditions. However, these disordered bilayers do not diffract well, i.e. they give rise to a limited number of quasi-Bragg peaks, and as such do not lend themselves ideally to traditional crystallographic analysis.

Data were thus collected in two different states of the membrane complexes: (1) at 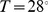
C and 50% relative humidity, in a de-hydrated gel state to emphasize the structural features in the X-ray scattering experiment (the location of the A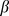 peptides was determined in this state); and (2) at 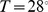
C and 100% relative humidity to ensure full hydration of the membranes and thus study structure in the fluid, physiologically relevant state of the membrane complexes. These fluid membranes were used to study the interaction between the A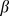 peptides and cholesterol and melatonin.

The results section is organized as follows: we first review structural properties of the A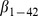 and A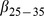 peptides in the next section before discussing partitioning of the A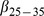 and A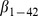 peptides in the DMPC/DMPS lipid matrix. We then present and discuss the experiments addressing the interaction between cholesterol plaques and A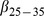 and the interaction between A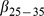 and melatonin.

### Structure of Amyloid-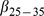 and Amyloid-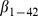


**Figure 2:**
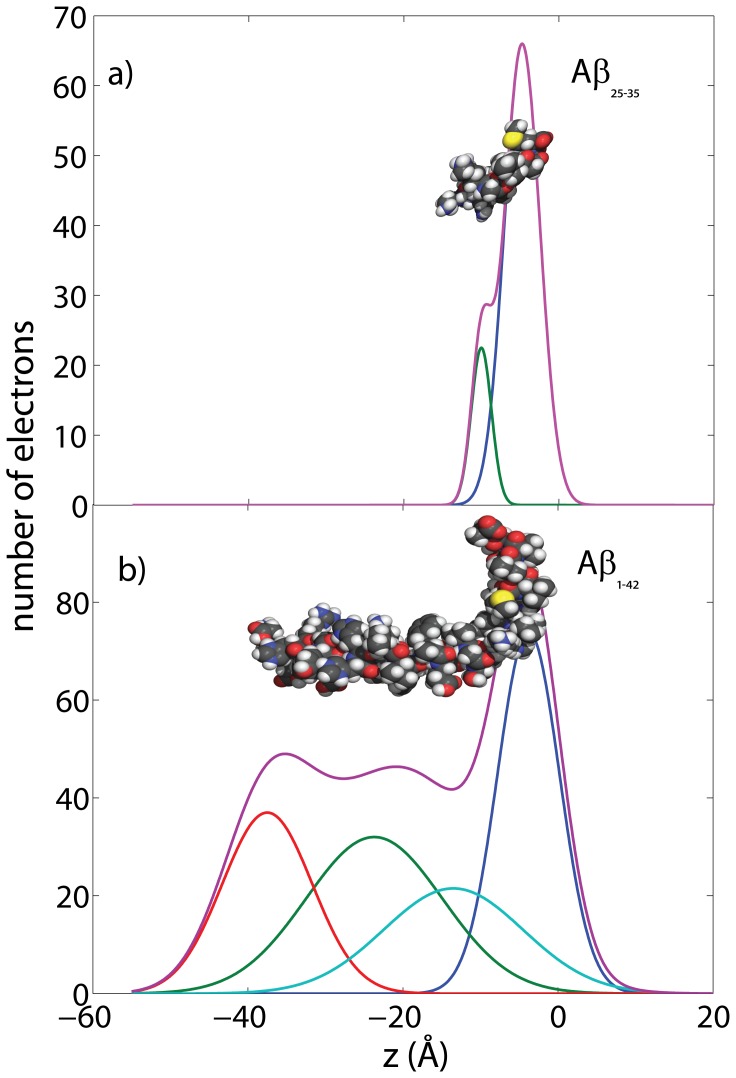
Calculated electron distribution of A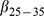 and A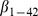. The molecular structures and calculations are based on the PDB structures 1QWP (A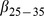) and 1IYT (A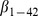). The peptides take helix-kink and helix-kink-helix configurations, respectively, in solution.

The solution structures of the A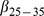 fragment (sequence ^25^G-S-N-K-G-A-I-I-G-L-^35^M) and the full length A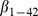 peptide were reported by D′Ursi *et al.* [66] and Crescenzi *et al.* [67] from nuclear magnetic resonance (NMR). The corresponding structure files are deposited in the Protein Database (PDB) as 1QWP (A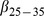) and 1IYT (A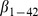). X-ray diffraction is sensitive to the electronic structure of bilayers and peptides. In order to compare the experimentally determined electronic profiles, the PDB structures were used to calculate 1-dimensional projections of the electron distributions along the 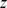-axis of the peptides. To take into account thermal motions of atoms and electrons, each atom was modeled by a Gaussian distribution with a width (FWHM) of 4 Å, and the corresponding electron distributions were summed. The results are plotted in Figures 2 a) and b).

The electron distribution of the A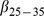 in Figure 2 a) was well fit by two Gaussian distributions. A sum of 4 Gaussians provided good agreement with the electron distribution of the A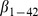 peptide, shown in part b). In both peptides, the Methionine at position 35 is discernable because of its electron rich sulphur atom. The comparison between the calculated distribution with the experimentally determined electron density will be used in the following section to determine the precise location and orientation of the two peptides in the anionic lipid bilayers.

### Interaction of Amyloid-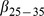 and Amyloid-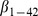 With Anionic Lipid Bilayers

**Figure 3:**
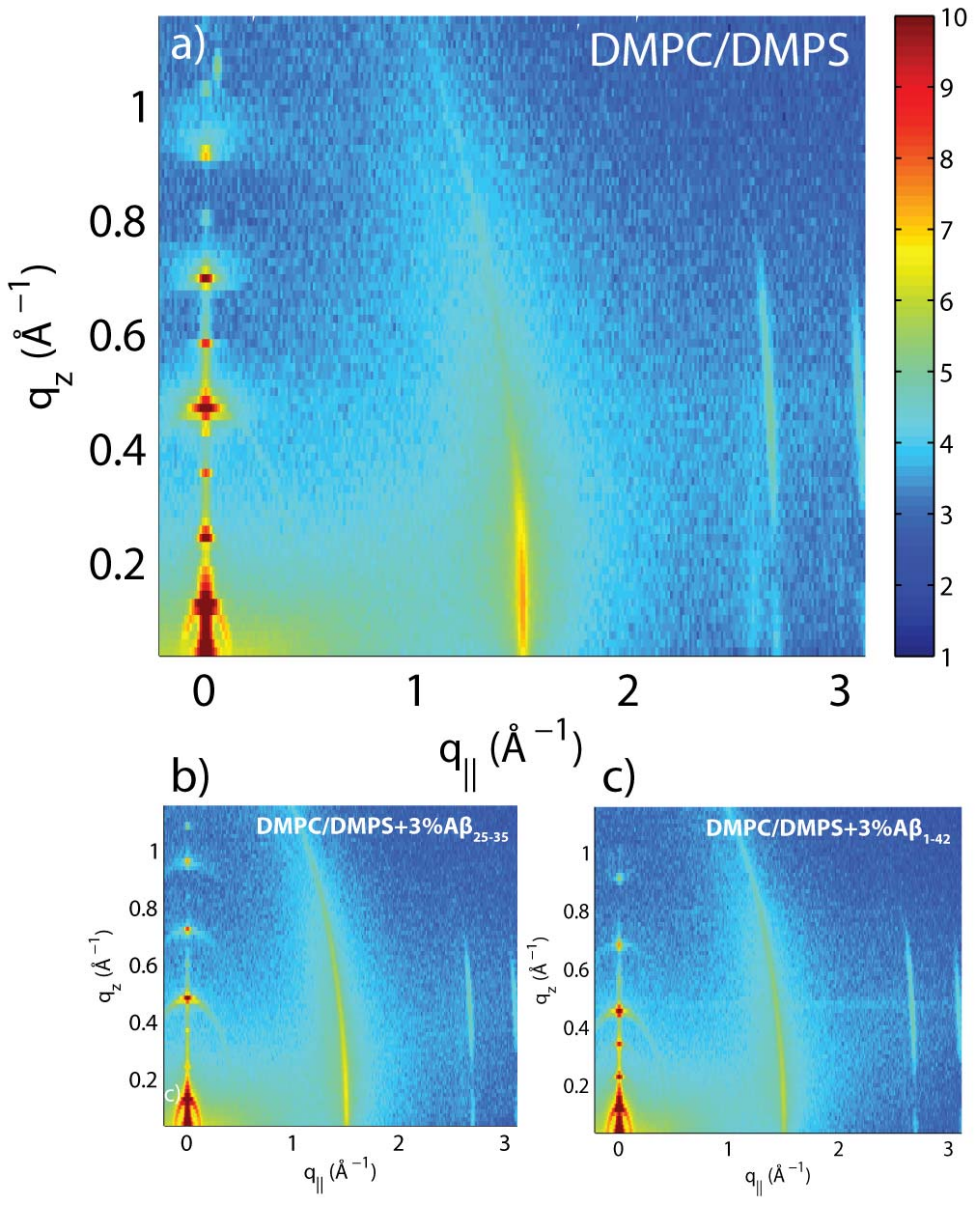
Two-dimensional data for (a) DMPC +3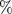 DMPS membrane, (b) DMPC/DMPS+3 mol% A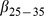 and (c) DMPC/DMPS+3 mol% A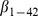. The area per lipid is obtained from the position of the lipid acyl chain correlation peak at 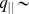 1.5 Å^−1^.

Figure 3 shows 2-dimensional X-ray intensity maps for a) DMPC/DMPS, b) DMPC/DMPS+3 mol% A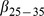 and c) DMPC/DMPS+3 mol% A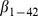. Data were collected at 50% relative humidity, in a de-hydrated gel state of the lipid bilayers, to enhance structural features and achieve a high spatial resolution, as demonstrated by Hristova and White [68]. The out-of-plane scattering along 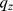 in Figure 3 shows pronounced and equally spaced Bragg intensities due to the multi-lamellar structure of the membranes, as reviewed for instance in [69], [70].

The diffracted intensity shows one well developed in-plane Bragg peak along the 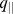-axis at 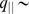
1.5 Å^−1^, related to the packing of the lipid tails. The intensity has a distinct rod-like shape, typical for a 2-dimensional system. The area per lipid molecule can be determined from the in-plane diffraction data, when assuming that the lipid tails form a densely packed structure with hexagonal symmetry (planar group p6), as reported from, e.g., neutron diffraction [55]. In the absence of fluctuations (in gel state lipid bilayers), the area per lipid molecule can be determined from the position of the in-plane Bragg peak at 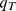 to 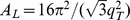 [56], [71], [72]. The distance between two acyl tails is determined to be 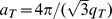, with the area per lipid simplified to 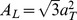, as listed in Table 1.

The area per lipid can be compared to results published by Tristram-Nagle, Liu, Legleiter and Nagle [73], who provide a reference for the structure of the gel phase in DMPC membranes. The authors find an area per lipid of 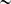
47 Å^2^ in fully hydrated bilayers at 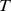 = 10°C. However, the observed area of 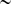
41 Å is still larger than the optimum packing for all-trans chains of about 40 Å^2^ [74], indicating that the overall lipid area is determined by the head group steric limit [75].

**Figure 4:**
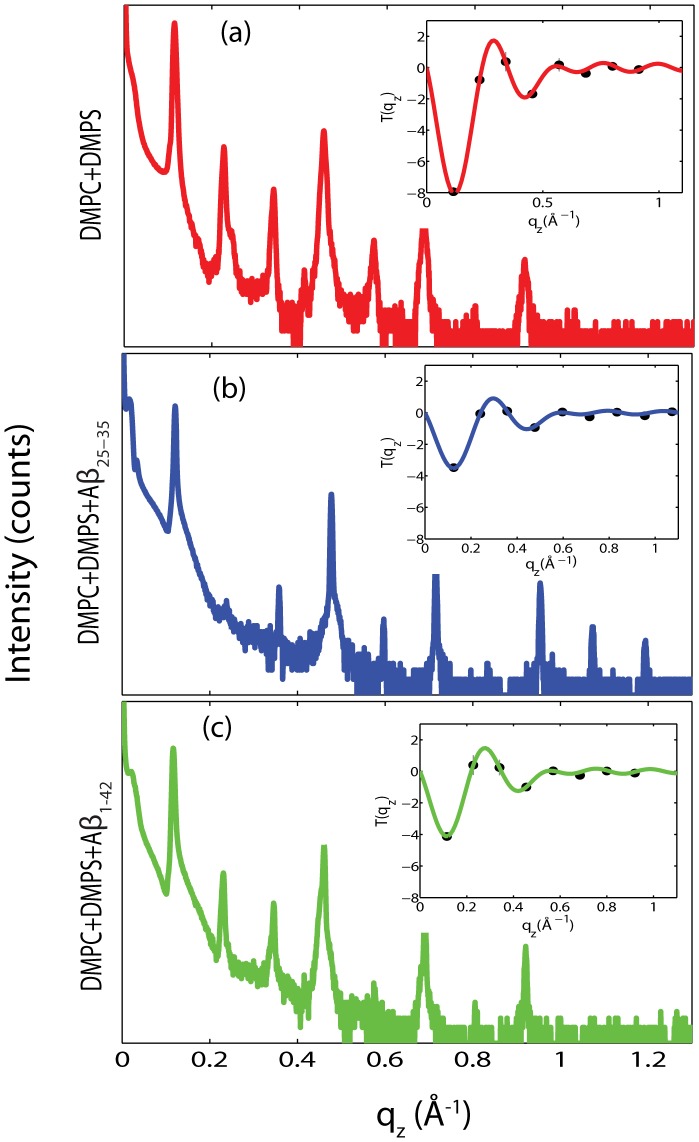
Reflectivity measurements for (a) DMPC/DMPS membrane, (b) DMPC/DMPS+3 mol% A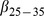, and (c) DMPC/DMPS+3 mol% A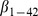. Pronounced and equally spaced Bragg peaks were observed, which are indicative to a well ordered lamellar structure. Electron densities were calculated through Fourier transformation of the integrated peak intensities. The insets shows the 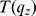 function, which was used to assess the phases of the corresponding Fourier components.

**Figure 5:**
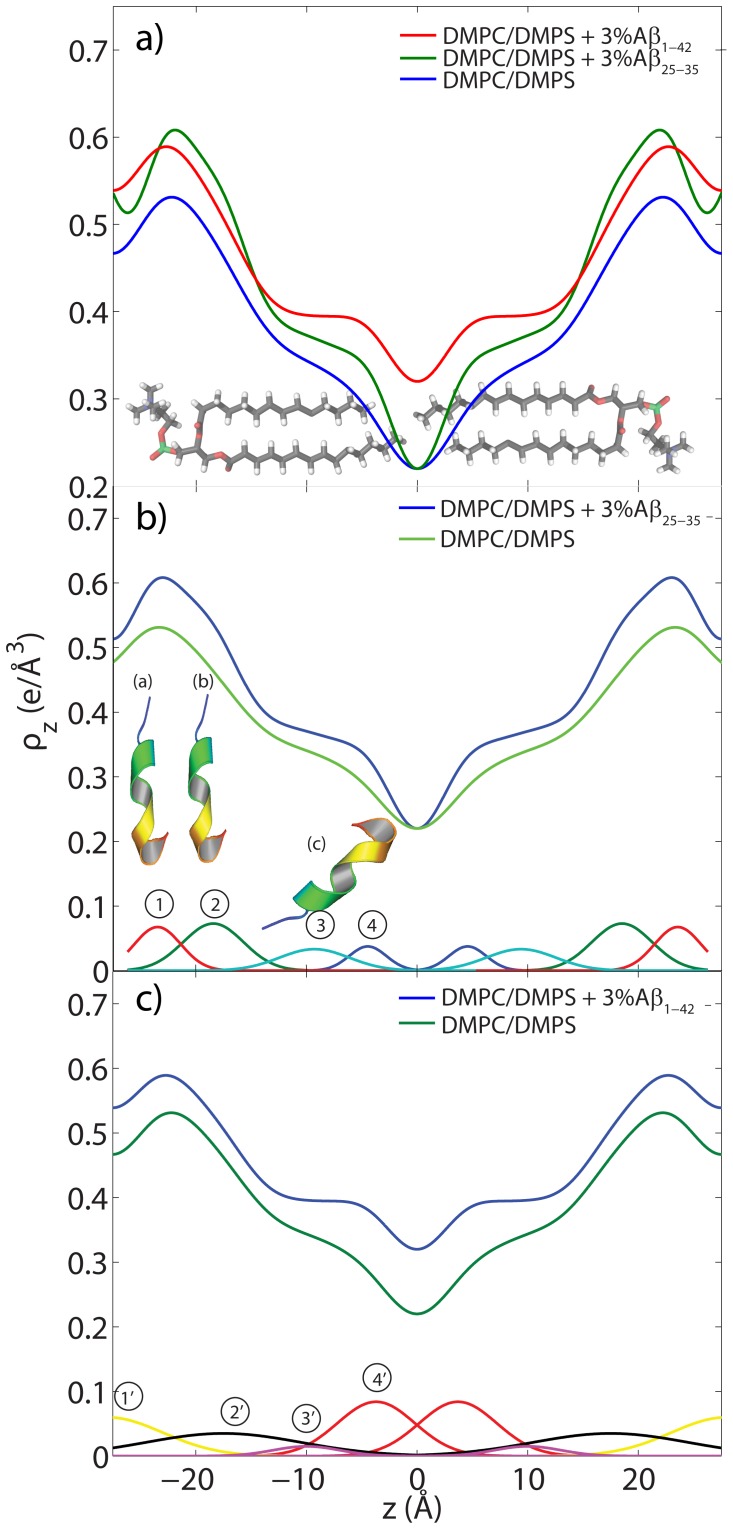
Electron density analyses obtained through Fourier transforms of the X-ray reflectivity data. a) Electron densities for the DMPC/DMPS membrane (green), DMPC/DMPS+3 mol% A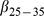 (blue), and DMPC/DMPS+3 mol% A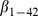 (red). b) The location of the A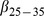 peptide in the DMPC/DMPS membrane, as determined from the difference electron density profile peak positions. c) Gaussian fits to the difference electron density obtained by subtracting the electron density profile of the DMPC/DMPS sample from that of the DMPC/DMPS+3 mol% A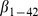 sample. A total of 4 peaks in each half of the bilayer are needed to fit the experimental data. The 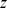-axis encompasses the full bilayer.

For a quantitative analysis of the diffracted intensity, the 2-dimensional data were cut along the out-of-plane and in-plane axes. The corresponding reflectivity curves are shown in Figure 4 a)-c). A series of pronounced and well developed Bragg peaks with up to 10 Bragg orders were observed, indicative of a well ordered lamellar structure. The lamellar spacings, 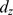, of the membrane complexes, i.e., the distance between two bilayers in the membrane stack, can be determined from the position of the Bragg peaks, and are listed in Table 1. The electron densities, 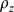, were determined from a Fourier transformation of the integrated peak intensities, as explained in the Materials and Methods Section. 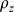 for DMPC/DMPS, DMPC/DMPS+3 mol% A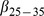, and DMPC/DMPS+3 mol% A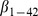 are shown in Figure 5. The profile for DMPC/DMPS in Figure 5 a) corresponds to a lipid bilayer in its gel state with both chains in all-trans configuration. The electron rich phosphorous group in the head group region can be identified by the peak in the electron density at 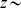
22 Å. 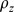 monotonically decreases towards the bilayer center at 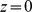; CH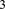 groups reside in the center with an electron density of 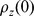 = 0.22 e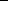/Å^3^ [56], [72], [76].

The electron densities in Figure 5 show an additional electron density in the presence of A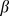 for both the A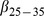 and the A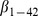 peptides, as shown in Figure 5 b) and 5 c), respectively. In order to determine the position of the peptides in the lipid matrix, the difference in electron density in the presence of the peptides was calculated. We note that this approach is strictly correct only under the assumption that the lipid matrix is not altered upon introduction of the peptide. Buchsteiner *et al.* [77] and Buchsteiner, Hauβ and Dencher [78] have shown that the structure of the membranes is unaffected at a low peptide concentration of 3 mol%, as used in our study. The difference between the electron density of the pure lipid matrix and the electron density in the presence of A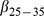 is plotted in Figure 5 b), and was well fit by 4 Gaussian peak profiles, 1, 2, 3, 4 (peak parameters are listed in Table 2). The number of electrons associated with the Gaussian distributions were calculated from the Gaussian area; these values as well as the areas per lipid molecule are stated in Table 2.

**Table 2:** Parameters for the Gaussian fits to the A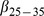 and A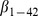 distributions.

A 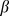	Gauss	Position	Width	Amp	Number of electrons	Fraction of peptide electrons
		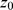	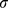	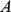		
(25–35)	1	22.0	5.0	0.07	17.2	29.5%
	2	17.6	2.0	0.06	17.7	30.5%
	3	9.0	2.0	0.03	16.0	27.5%
	4	4.3	2.0	0.03	7.2	12.5%
					58.2	100%
(1–42)	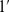	28.0	5.0	0.06	27.1	25.5%
	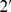	17.5	7.0	0.04	32.5	30.6%
	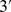	10.0	3.0	0.02	6.2	5.8%
	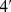	3.7	3.5	0.08	40.6	38.1%
					106.4	100%

The number of electrons is calculated by integrating across the Gaussian peaks, and the fraction is calculated by dividing by the total number of peptide electrons.

**Figure 6:**
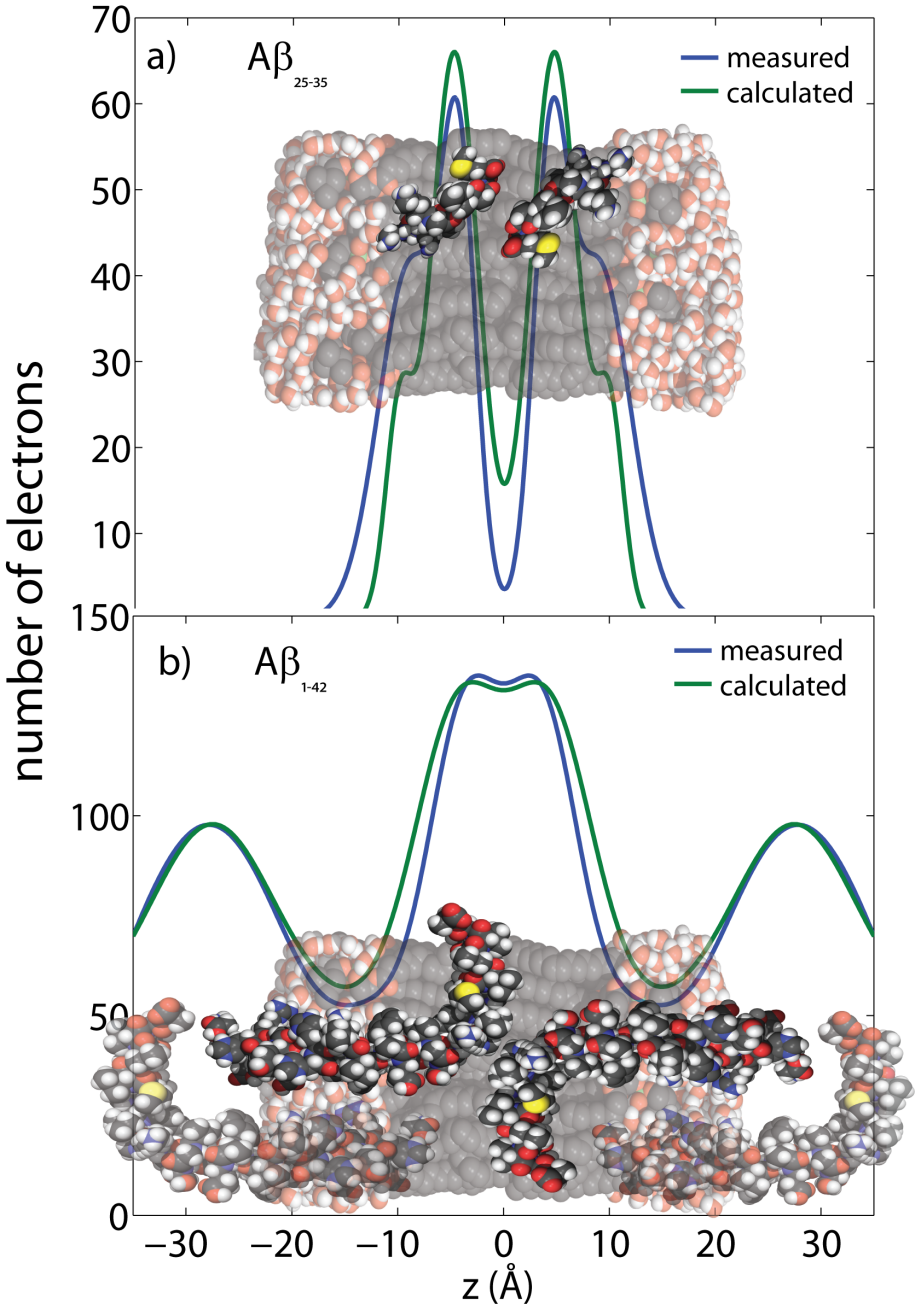
Measured and calculated electron distribution of the membrane-embedded A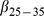 a) and A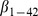 b) peptides and their position in the membrane. Good agreement between calculations and experiments is obtained for a position of A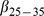 in the hydrocarbon membrane core. The peptide takes a slightly tilted orientation, in agreement with computer simulations [85]. The full length A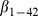 peptide was also found to embed in anionic lipid exclude a membrane-spanning 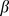-sheet structure, as it was reported from molecular dynamics simulations [82]–[84].

The experimentally determined electron density can be compared to the electron distribution calculated from the PDB data in the Section: *Structure of Amyloid-* 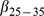 *and Amyloid-* 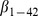. Distributions 3 and 4 agree well with the electron density calculated for the A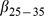 fragment. The A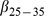 peptides were found to embed in the hydrophobic membrane core at 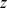 values of 2 Å
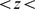
18 Å, as shown in in Figure 6 a). The percentage occupation of this position can be calculated by comparing the number of electrons associated with peaks 3 and 4 to the total number of peptide electrons. 40% of the A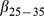 peptides were found to be embedded in the anionic bilayers. Distributions 1 and 2 were assigned to membrane-bound peptides at 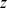 positions of 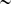
18 Å and 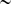
22 Å, in the head group region of the bilayers. These peptides did not penetrate the membrane core, however aligned themselves in the plane of bilayers. About 30% peptides were found in membrane-bound state 2 and 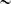
30% of the peptides were found in position 1, at the head group to water interface.

The difference in the electron density in the presence of the full length A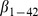 peptide in Figure 5 c) was well fit by 4 Gaussian distributions (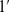, 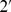, 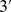, 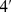). The comparison between the electron density calculated in the Section: *Structure of Amyloid-* 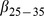 *and Amyloid-* 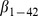 and the experimentally determined electron density is shown in Figure 6 b). The peptide was translated along the 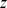-axis and rotated until the calculated and experimental electronic distributions showed a good agreement. As the experimental data are symmetric and periodic, the profiles of two peptides in the two different leaflets, and also peptides from neighboring bilayers were included in the calculation, as displayed in the Figure. The full length A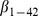 peptide was found to embed in anionic DMPC/DMPS bilayers. We note that insertion of 3 mol% A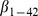 in the DMPC/DMPS membranes did not change the lamellar 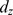-spacing, as listed in Table 1. This is very likely an effect of the reduced hydration of the membrane complexes used. The effect of cholesterol on the position of the A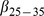 peptide will be studied in the next section.

### Interaction of Amyloid-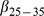 With Immiscible Cholesterol Plaques

**Figure 7:**
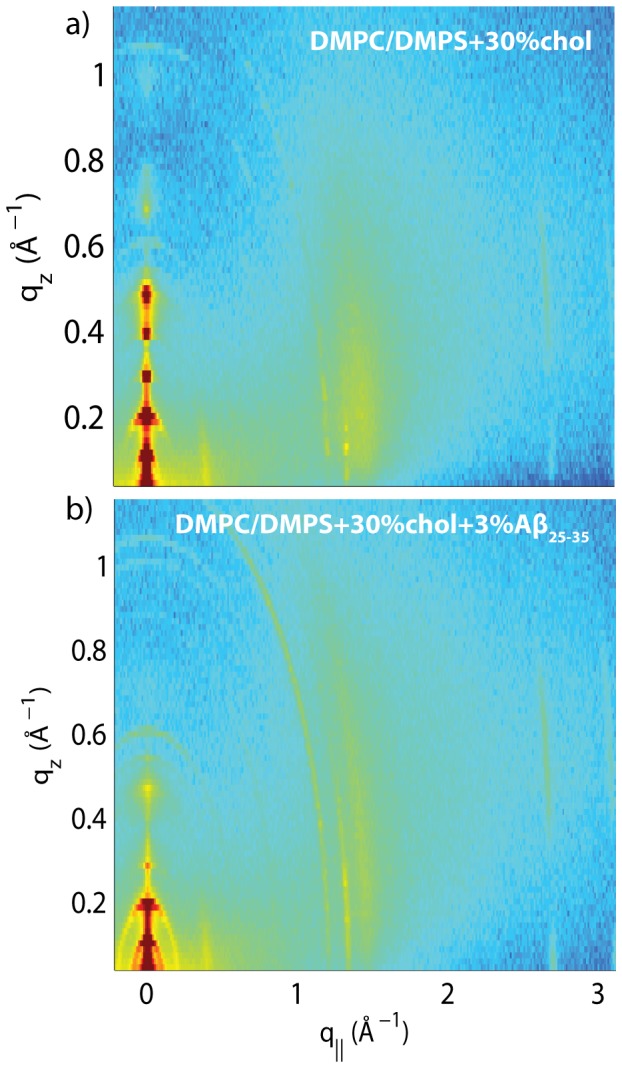
Two-dimensional X-ray scans for the a) DMPC +3 mol% DMPS +30 mol% cholesterol and b) DMPC +3 mol% DMPS 3 mol% A
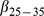
+30 mol% cholesterol membranes. Data were collected under full hydration of the membranes (100% RH), in their physiologically relevant fluid state.

Figure 7 shows 2-dimensional scans for a) DMPC/DMPC+30 mol% cholesterol and b) DMPC/DMPC+30 mol% cholesterol+3 mol% A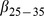 collected under full hydration of the membranes (100% RH). While the pronounced feature along the 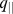-axis of the pure DMPC/DMPS bilayers (Figure 3) is the lipid acyl chain correlation peak at 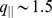 Å^−1^, the addition of cholesterol in Figure 7 a) led to the occurrence of additional rod-like scattering intensities at 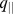-values of 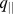 = 1.19 Å^−1^ and 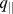 = 1.32 Å^−1^, as listed in Table 3. The fact that extra intensities were observed along the in-plane axis, yet were not observed along the out-of-plane axis indicates that the molecular packing in the membrane plane changed at this composition; however, the topology of the multi-lamellar assembly remained unaltered. The DMPC/DMPS+30 mol% cholesterol+3 mol% A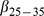 membranes in Figure 7 b) show an increase in intensity of the in-plane cholesterol features shown in a).

**Table 3:** Peak parameters for the fits to the in-plane cholesterol data shown in Figure 8.

Sample	Peak	Position	Width	Amplitude	Area
		(Å 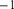 )	(Å 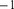 )		(counts 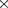 Å 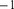 )
DMPC/DMPS	[110] 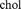	1.19	0.02	1460	73
+30 mol%chol	[200] 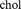	1.32	0.02	2500	98
	[100] 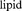	1.42	0.13	3700	1216
DMPC/DMPS	[110] 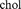	1.18	0.02	1450	63
+30 mol%chol	[200] 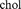	1.32	0.02	3880	158
+3 mol%A 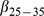	[100] 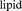	1.45	0.09	2370	552

The [110] and [200] reflections are well fit by a monoclinic lattice with lattice parameters of 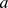 = 9.76 Å, 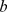 = 7.56 Å and 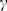 = 103.

**Figure 8:**
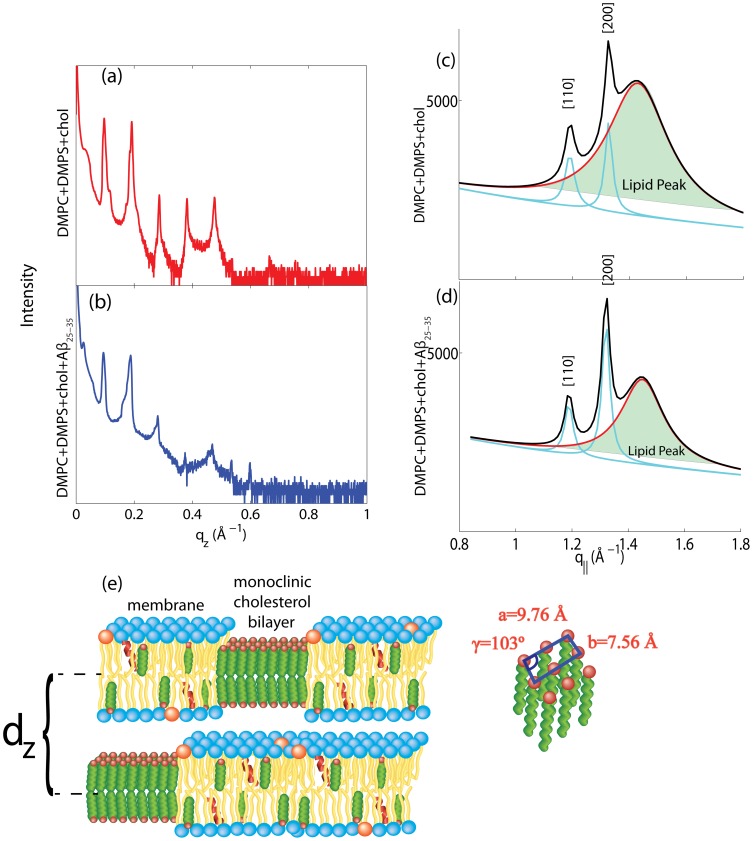
Reflectivity measurements for the a) DMPC/DMPS+30 mol% cholesterol membrane and the b) DMPC/DMPS+3 mol% A
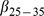
+30 mol% cholesterol membrane. c) and d) show Lorentzian fits to corresponding in-plane data from Figures 7 c and d), scanned under 100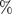 relative humidity. Note that Barrett *et al.* [56] identified the blue peaks as corresponding to in-plane features of crystalline cholesterol plaques. e) Cartoon of the structure of the multi-lamellar membrane complexes with membranes and coexisting cholesterol plaques and corresponding 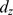-spacing. The cholesterol molecules in the plaques form a monoclinic lattice.

The lamellar 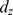-spacing was determined from X-ray reflectivity curves at full hydration for DMPC/DMPC+30 mol% cholesterol and DMPC/DMPC+30 mol% cholesterol with 3 mol% A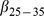, as shown in Figure 8 a) and b).

The formation of immiscible cholesterol plaques in model membranes made of DMPC was reported recently by Barrett *et al.* [56]. These plaques were found to consist of cholesterol bilayers coexisting with the lamellar membrane phase, as depicted in Figure 8 e). The cholesterol molecules in these plaques were found to be highly ordered and form a monoclinic lattice, leading to the additional in-plane signals in the 2-dimensional data. The in-plane peaks in Figures 8 c) and d) agree well with the [110] and [200] reflections of a monoclinic lattice with lattice parameters of 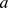 = 9.76 Å, 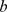 = 7.56 Å and 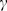 = 103°, as observed by Barrett *et al.* [56].

Addition of the A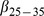 peptide has two effects on the membrane structure: the monoclinic in-plane peaks increase in intensity by 30%, while the integrated intensity of the lipid peak decreases by 65%. In addition, higher order reflectivity Bragg peaks were strongly suppressed in Figure 8 b), in the presence of A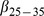, typical for an increase in fluidity of the membranes. The most likely structure of the membrane system is shown in Figure 8 e). Barrett *et al.* observed a coupling between the cholesterol bilayers in neighboring bilayers in the stack, which led to the occurrence of a second 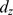-spacing and the split of the reflectivity peaks. The observation of a single 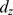-spacing is indicative that the cholesterol plaques are randomly distributed throughout the membrane stack.

The increase in the in-plane cholesterol peak intensities points to an increased volume fraction of cholesterol plaques in the presence of the peptides. The most likely explanation is that the peptides strongly associate with the lipid bilayers and displace cholesterol molecules, which then form additional cholesterol bilayers. We note that the 1-dimensional electron densities in the presence of lateral membrane plaques are superpositions of the two coexisting structures. It is, therefore, not possible to unambiguously determine the position of the peptides in the bilayers in the presence of cholesterol plaques from X-ray reflectivity.

### Interaction of Amyloid-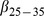 With Anionic Lipid Bilayers Containing Melatonin

**Figure 9:**
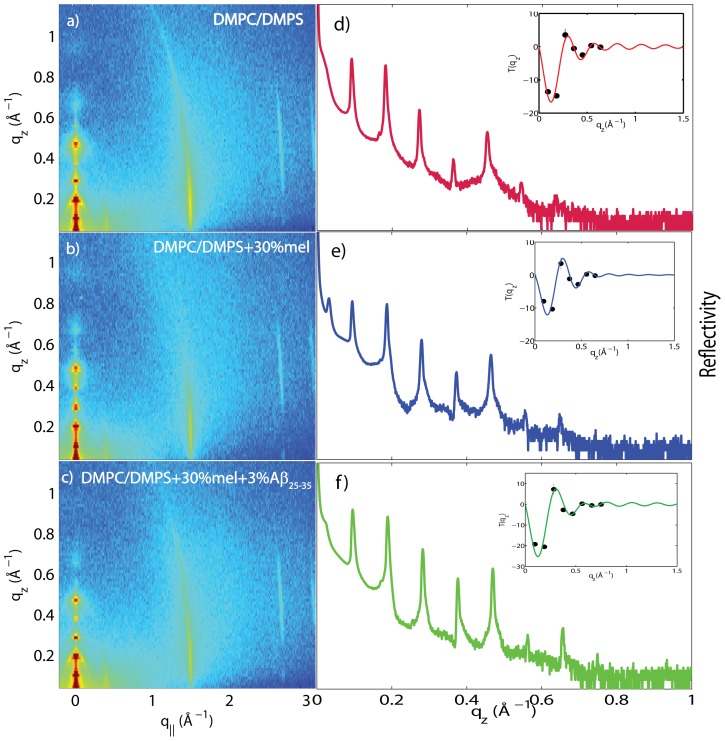
Two-dimensional X-ray data for the a) DMPC/DMPS, b) DMPC/DMPS +30 mol% melatonin, and c) DMPC/DMPS +30 mol% melatonin +3 mol% A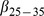 samples, with all scans done under full hydration. Corresponding reflectivities are shown in d), e) and f). The phases can be determined from the 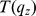 function (shown as insets to the plots) and were used to determine the electron densities plotted in Figure 10.

**Figure 10:**
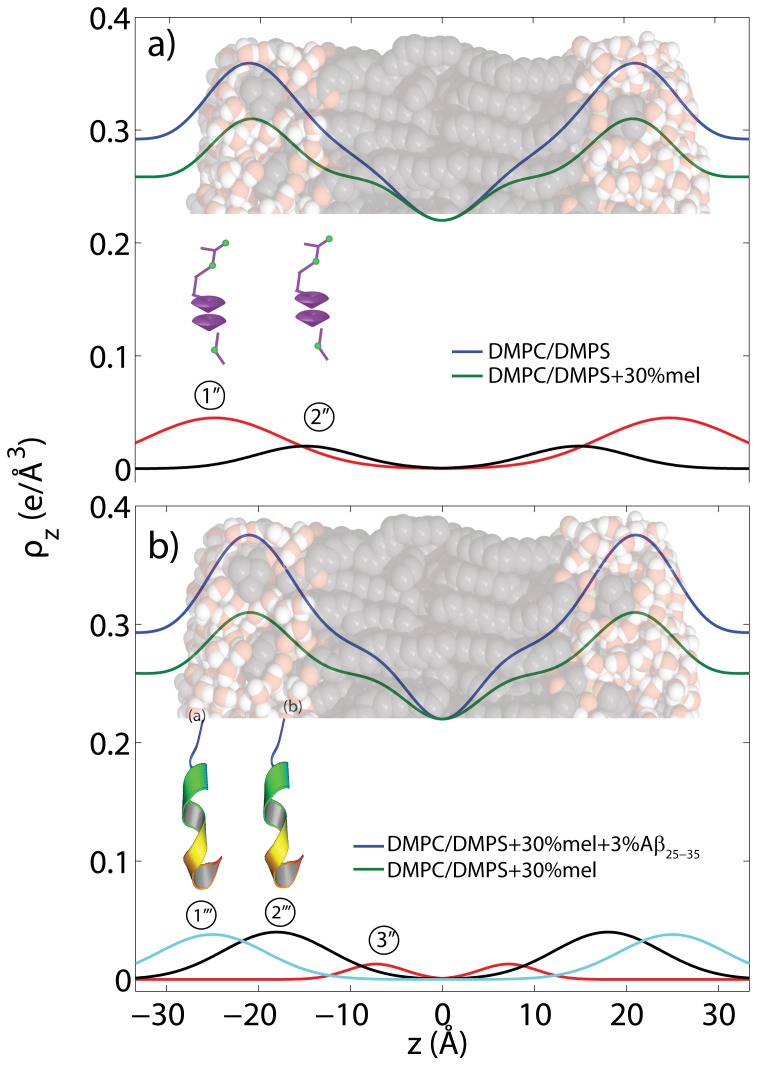
Electron densities for the a) DMPC/DMPS, b) DMPC/DMPS +30 mol% melatonin, and c) DMPC/DMPS +30 mol% melatonin +3 mol% A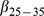 samples. All samples were scanned under 100% RH. The difference in electron density in a) allows the determination of position of the melatonin molecules in the anionic lipid membranes. The location of the A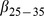 peptide in the DMPC/DMPS+30 mol% melatonin membrane was determined from the difference electron density profile peak positions in b).

The addition of melatonin to the DMPC/DMPC bilayers does not lead to additional in-plane or out-of-plane signals, as can be seen in the 2-dimensional data in Figures 9 a), b) and c) for DMPC/DMPS, DMPC/DMPS +30 mol% melatonin and DMPC/DMPS+30 mol% melatonin +3 mol% A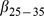. Well-defined reflectivity curves could be measured and are shown in Figures 9 d), e) and f). The data in Figure 9 were measured under full hydration of the membranes, in a 100% H
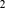
O atmosphere. The lamellar 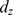-spacing for DMPC/DMPS at 100% relative humidity of 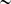
69 Å can be compared to values of 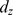 for pure DMPC bilayers as a function of relative humidity published by the Nagle group [79]; this value indicates that the membranes were in their fully hydrated fluid phase. The corresponding electron densities are displayed in Figure 10. The location of the melatonin molecules in the bilayers and their impact on peptide positions were determined from these measurements.

We note that it is not straightforward to determine lipid areas from the position of the acyl chain correlation peak in the fluid phase of the bilayers, due to the effect of strong fluctuations in this state of the membranes. The development of experimental techniques to determine fluid phase lipid areas by a combination of X-ray and neutron diffraction and computer modelling by Kučerka *al.* [80] was a milestone in the quantitative determination of material parameters in model biomembranes [81]. As fluid phase areas have not yet been published for the membranes in this study, we used an area of 60.6 Å^2^, determined for fluid DMPC bilayers by Kučcerka *et al.* [79].

The positions of the melatonin molecules were determined from the difference in electron densities between DMPC/DMPS and DMPC/DMPS+30 mol% melatonin membranes, as shown in Figure 10 a). As reported recently by Drolle *et al.* [31], the melatonin molecules were found to reside in the lipid head group region. The electron density gives evidence for 2 populations: population 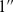 at the membrane-water interface (at 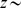
25 Å) and population 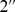 (at 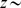
15 Å) at the interface between the hydrophilic head group region and the hydrophobic hydrocarbon chain core. Based on the area of the corresponding contributions, 74% of the melatonin molecules are found at the water interface, 26% at the hydrophobic membrane core interface. We note that the electron density reduces when melatonin molecules are incorporated because electron-rich phospholipid molecules (see Table 4) are replaced by the smaller melatonin molecules. The Gaussian distributions in Figure 10 a) are, therefore, indicative of a lack of electron density when compared to the DMPC/DMPS bilayer.

**Table 4:** Composition and number of electrons of the different molecular components.

Molecule	Chemical Formula	M 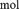 (g/mol)	# of 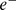
DMPC	C 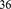 H 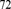 NO 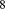 P	677.9	374
DMPS	C 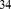 H 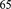 NO 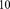 PNa	701.8	372
cholesterol	C 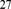 H 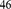 O	384.7	216
melatonin	C 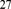 H 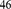 O	232.3	124
water	H 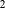 O	18.0	10
A 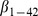	C 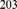 H 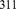 N 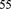 O 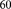 S	4514.1	2410
A 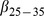	C 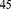 H 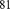 N 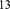 O 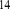 S	1060.3	678

Figure 10 b) shows the difference between the electron densities of DMPC/DMPS +30 mol% melatonin +3 mol% A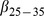 and DMPC/DMPS +30 mol% melatonin. The electron distribution can be well fit by 3 Gaussian distributions and assigned to 3 peptide populations, as shown in Figure 10 and listed in Table 5. When compared to the position of the A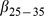 in pure DMPC/DMPS bilayers, the fraction of membrane-bound peptides was found to increase in the presence of melatonin. About 46% of A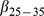 resides at the membrane water interface, with 46% at the interface between the hydrophilic head group region and the hydrophobic hydrocarbon core region. Distribution 3′′ is indicative of a small fraction of membrane embedded peptides.

**Table 5:** Parameters for the Gaussian fits to the A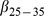 distributions for the melatonin-containing membrane.

A 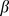	Gauss	Position	Width	Height	Number of electrons	Fraction of peptide electrons
		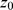	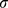	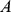		
(25–35)	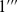	25	5.8	0.038	35.15	46%
	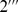	18	5.8	0.040	35.15	46%
	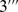	7.2	3.1	0.013	6.06	8%
					76.36	100%

The number of electrons is calculated by integrating across the Gaussian peaks, and the fraction is calculated by dividing through by the total number of peptide electrons.

In agreement with Drolle *et al.*, the addition of 30 mol% melatonin led to a decrease in the lamellar 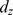-spacing from 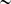
69 Å to 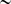
67.5 Å. The addition of 3 mol% A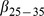 was found to further decrease 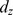 to 66.7 Å. The decrease in 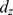 is accompanied by a slight increase in 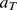 (the distance between two neighboring lipid tails) from 4.87 Å to 4.89 Å is compatible with an increase in fluidity of the membranes, as suggested by Drolle *et al.*

## Discussion

### Position of Amyloid-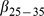 and Amyloid-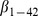 in Anionic Lipid Membranes

The positions of the full length A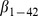 and the A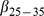 fragment in gel state anionic lipid bilayers made of DMPC and DMPS were determined by a combination of electron density measurements and electronic structure calculations based on PDB structure files. Good agreement between calculations and experiment was obtained for an embedded state of the A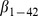 peptide. In addition to two membrane-bound states, the A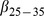 segment was found to embed in the hydrophobic membrane core.

A membrane-embedded state of the A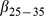 peptide was previously reported by Mason *et al.* [21] and Dante, Hauβ and Dencher [11]. Evidence for a membrane-embedded state of the A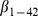 peptide was presented previously by Dante *et al.* [22]. Several conclusions can be drawn from our X-ray diffraction experiments: Addition of 3 mol% A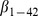 or A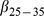 to the stacked anionic DMPC/DMPS membranes did not change the topology of the membrane stack or disrupt the lamellar structure, as evidenced by the well developed reflectivity curves in Figure 4. From the absence of additional in-plane signals in the 2-dimensional data in Figure 3 the lateral membrane structure was conserved with no signs of the formation of pores or other membrane defects.

While A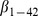 forms a helix-kink-helix structure in solution [67], 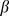-sheet structures were reported in oligomers or senile plaques [82], most likely induced through peptide-peptide and lipid-peptide contacts. From the agreement between calculated and measured electron distribution in Figure 6 b) we do not find evidence for a membrane-spanning 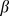-sheet structure, as was reported from molecular dynamics simulations [82]–[84]. This is most likely related to the low peptide concentration of 3 mol%. We also did not detect membrane-bound A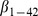 peptides in this experiment using incubated membranes. The position at the hydrophilic interface of the bilayers is most likely less favorable in stacked membranes than in single solid supported membranes, where surface bound A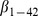 peptides were observed using AFM [17], [34].

Three populations were found for the A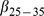 fragment. After 15 hours of incubating in a 100% water atmosphere at 30°C, 40% of the A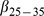 peptides were found to embed into the anionic lipid bilayers, at 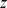 positions of 2 Å
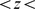
18 Å. The peptides were found to take a slightly tilted position in the bilayers, where the fragment is oriented similarly as the 25–35 segment in the full length peptide A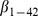. Based on the good agreement between experimental electron distribution, A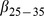 peptides most likely form a helical structure when embedded in membranes, as depicted in Figure 6. In agreement with the molecular dynamics simulations by Tsai *et al.* [85], we did not find evidence for a 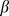-hairpin confirmation, which may play a significant role in initiating peptide aggregation.

In addition to the embedded state, the electron densities provide evidence for two membrane-bound states in the hydrophilic head group region, where the peptides align parallel to the membrane surface. 30% of the A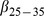 peptides were observed at a 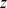-value of 18 Å, at the interface between the hydrophilic head group region and the hydrophobic chain region. 30% of the peptides were found at the membrane-water interface, at 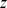 = 23 Å. These membrane-bound peptides can very likely be observed in AFM experiments on solid supported membranes [17]. The experimental findings of membrane-bound and embedded states are in agreement with results from computer modeling by Tsai *et al.* [85]. The peptides were found to align parallel to the membranes at the lipid-water interface in the simulations.

The interaction between peptides and membranes is often modelled by the two-stage or two state-model [86]–[88]. In a first step, the peptide makes contact with the membrane and aligns parallel to the membrane, before stronger bonds form and the peptide is embedded into the hydrocarbon core. The population ratio between surface-bound and embedded states directly provides the free energy of insertion 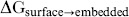 [89]. At 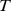 = 28°C, we found 60% of the A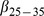 peptides to be surface bound, in two different positions in the head group region, and 40% of the peptides to be embedded in the hydrocarbon membrane core.

### Peptide Interaction With Immiscible Cholesterol Plaques

Cholesterol, a steroid molecule with a hydrophilic head and a hydrophobic tail, is known to insert itself into lipid bilayers [53], [55], [69], [90]–[92]. In saturated lipid bilayers, the cholesterol molecules align parallel to the lipid acyl chains, an arrangement that is well-known as the umbrella-model [93], [94]. The addition of cholesterol to fully hydrated lipid membranes usually leads to a significant decrease in the area per lipid molecule. This is the result of cholesterol's condensation effect, i.e., the suppression of fluctuations and the ordering of a lipid's hydrocarbon chains [95], [96].

It was recently shown that the liquid ordered, 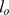, phase of lipid membranes is not uniform, but consists of lipid domains, which are saturated with cholesterol, in equilibrium with a disordered fluid membrane [53]–[55], [92], [97], [98].

Cholesterol plaques are speculated to have significance in early stages of atherosclerosis, as precursors of atherosclerotic plaques [63]. The DMPC/DMPS/cholesterol system provides a good model system to study the interaction between A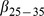 with immiscible cholesterol plaques. The occurrence of immiscible cholesterol plaques in binary DMPC/cholesterol model membranes at high cholesterol concentrations was previously reported by Barrett *et al.* [56], and the ordering of the cholesterol molecules was determined. Formation of immiscible cholesterol plaques in DMPC/cholesterol membranes was reported to occur at cholesterol concentrations of 40 mol%. As cholesterol plaques were observed at 30 mol% cholesterol in Figure 8, the addition of DMPS was found to significantly lower the solubility limit of cholesterol in saturated lipid membranes.

The addition of 3 mol% A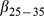 was found to lead to an increase in the total fraction of cholesterol plaques in the stacked membranes. However, the monoclinic structure of the cholesterol molecules in these plaques did not change, indicating that A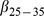 preferably interacts with the lipid domains. This is in agreement with the literature, where A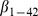 was reported to have a higher affinity for fluid membranes than for the high cholesterol (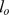) domains [19]. Dante, Hauβ and Dencher [12] have shown that small amounts of cholesterol (20 mol%) inhibit the insertion of A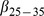 into fluid phase membranes. A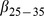 was found to be unable to intercalate into the membrane bilayer at high cholesterol content [99]. Microscopic observations of the membrane surfaces have shown an enhancement in phase separation of lipids as a result of interactions between peptides during induced aggregation of A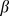 [100]. We note that the displacement of cholesterol molecules from the membranes into the immiscible cholesterol plaques also led to a decrease of cholesterol concentration and an increase of fluidity of the lipid bilayers, as shown in Figure 8.

### Effect of Melatonin

We determined the position of melatonin in fluid anionic DMPC/DMPS bilayers under full hydration. The hydrophilic melatonin molecules were found to reside in the hydrophilic head group region. The electron densities in Figure 10 a) provide experimental evidence for two populations: one at the membrane-water interface and a second population at the interface to the hydrophobic hydrocarbon chain region. This is in agreement with the results by Drolle *al.* for DPPC bilayers containing 9 mol% melatonin.

While the A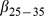 peptide was found in three locations in pure anionic DMPC/DMPS bilayers, the data in Figure 10 b) show that the population of the membrane embedded state is drastically reduced in the presence of melatonin. This is a remarkable observation, as the melatonin molecules seem to compete for space in the head group region with the peptides, yet they displace the peptides from the hydrocarbon core of the membranes. This displacement is most likely an entropic effect created by an increase in lipid hydrocarbon chain fluctuations due to the increase in membrane fluidity. This increased fluidification of the membranes may inhibit peptide insertion, in agreement with the previous observation that A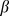 preferably interacts with gel phase membranes [33].

Age-related changes in melatonin [5]–[8] levels have previously been linked to the development of Alzheimer's disease. Our data show that a reduction in melatonin levels may reduce the number of surface-bound A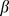 peptides and lead to an increase of membrane-embedded peptides. Penetration of peptides into the membrane core may facilitate oligomerization or fibrillation, as suggested by the two-stage model [86]–[88].

## Conclusions

We present experimental evidence for an interaction between amyloid-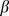 peptides with melatonin and cholesterol in anionic lipid membranes. The three important findings of this work can be summarized as follows. We present experimental evidence that the full length peptide embeds in the hydrocarbon core of anionic lipid bilayers. Three populations were found for the A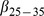 fragment: two membrane-bound states in the hydrophilic head group region of the bilayers, and one embedded state in the center of the membranes. These results shed new light on the location and environment of A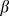 peptides, both of which are crucial to their interactions and potential aggregation behavior.

There is increasing evidence for a link between neurodegenerative diseases, such as Alzheimer's disease, and age-related changes in cholesterol and melatonin levels in brain tissue. Immiscible cholesterol plaques, i.e., cholesterol bilayers coexisting with the lamellar membranes, were created by addition of 30 mol% cholesterol to the anionic membranes. These plaques are precursors of atherosclerotic plaques and are good model systems to study age-related effects on membrane-peptide interactions. In the presence of immiscible cholesterol plaques, the A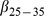 peptide was found to interact with the membrane component. The peptides were found to displace cholesterol molecules, leading to a reduced cholesterol concentration in the lipid regions and an increase in the total fraction of cholesterol plaques.

Melatonin molecules were found to reside at the head group-water interface and the interface between the hydrophilic head groups and the hydrophobic hydrocarbon chains. Addition of 30 mol% melatonin molecules to the anionic membranes was found to drastically reduce the population of the membrane-embedded A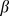 state.

## Materials and Methods

### Preparation of the Highly-Oriented Multi-Lamellar Membrane Samples

Highly oriented multi-lamellar membranes were prepared on single-side polished silicon wafers. 100 mm diameter, 300 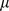
m thick silicon (100) wafers were pre-cut into 1
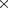
1 cm^2^ chips. The wafers were first pre-treated by sonication in dichloromethane (DCM) at 310 K for 25 minutes to remove all organic contamination and leave the substrates in a hydrophobic state. Each wafer was thoroughly rinsed three times by alternating with 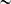
50 mL of ultra pure water and methanol.

Individual solutions of 1,2-dimyristoyl-sn-glycero-3-phosphocholine (DMPC), 1,2-dimyristoyl-sn-glycero-3-phospho-L-serine (DMPS), cholesterol, and melatonin (molecular representations are depicted in Figure 1 a)) were each dissolved in a 1∶1 chloroform:2,2,2-trifluoroethanol (TFE) solution at a concentration of 15 mg of lipid per ml solvent. The amyloid-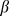 peptides were prepared by pretreatment with trifluoroacetic acid (TFA) to disaggregate the peptide, as described in [101]. This pretreatment included dissolving the peptide in a 1 mg/ml solution of TFA, sonicating with a tip sonicator for three five second intervals, and then removing the solvent through evaporation for 12 hours under vacuum at 298 K. The peptide was then redissolved in a 15 mg/ml solution of 1∶1 TFE:chloroform. The DMPS proved somewhat difficult to dissolve, necessitating several centrifugations in a Vortex mixer, as well as short periods of heating to 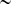
313 K in a hot water bath. The DMPS, DMPC, cholesterol, melatonin and peptide solutions were then mixed in appropriate ratios to produce the desired membrane samples for the experiment.

The tilting incubator was heated to 313 K and the lipid solutions was placed inside to equilibrate. 65 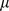
L of lipid solution was applied on each wafer, and the solvent was then allowed to slowly evaporate for 10 minutes at a speed of 15, tilt of 1, such that the lipid solution spread evenly on the wafers. After drying, the samples were placed in vacuum at 313 K for 12 hours to remove all traces of the solvent. The hydration container was allowed to equilibrate at 293 K in an incubator. The temperature of the incubator was then increased gradually from 293 K to 303 K over a period of 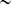
5 hours to slowly anneal the multi-lamellar structure. All samples were incubated for 15 hours at 303 K in a 100% water atmosphere before the measurements. This procedure results in highly oriented multi-lamellar membrane stacks and a uniform coverage of the silicon substrates. About 3,000 highly oriented stacked membranes with a thickness of 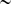
10 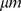 are produced using this protocol. The high sample quality and high degree of order is necessary to determine in-plane and out-of-plane structure of the membranes and the position of the cholesterol molecules. Table 1 lists all samples prepared for this study.

### X-ray Scattering Experiment

Out-of-plane and in-plane X-ray scattering data was obtained using the Biological Large Angle Diffraction Experiment (BLADE) in the Laboratory for Membrane and Protein Dynamics at McMaster University. BLADE uses a 9 kW (45 kV, 200 mA) CuK-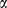 Rigaku Smartlab rotating anode at a wavelength of 1.5418 Å. Both source and detector are mounted on movable arms such that the membranes stay horizontal during the measurements. Focussing multi-layer optics provides a high intensity parallel beam with monochromatic X-ray intensities up to 10^10^ counts/(s
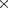
mm^2^). This beam geometry provides optimal illumination of the solid supported membrane samples to maximize the scattering signal. A sketch of the scattering geometry is shown in Figure 1 b). By using highly oriented membrane stacks, the in-plane (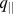) and out-of-plane (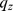) structure of the membranes could be determined. From the high resolution X-ray diffraction experiments we determine the molecular structure of the membranes in two different ways: (1) the out-of-plane membrane structure to determine the location of the different molecules in the membrane with sub-nanometer resolution and (2) the lateral organization of the different molecular components in the plane of the membrane. The result of such an X-ray experiment is a 2-dimensional intensity map of a large area (0.03 Å
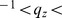
1.1 Å^−1^ and 0 Å
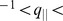
3.1 Å^−1^) of the reciprocal space. Data were collected at 28

C and 50% RH, in a de-hydrated gel state of the bilayers, and at 100% RH, in their fluid state.

### Out-Of-Plane Structure and Electron Densities

The out-of-plane structure of the membranes was determined using specular reflectivity, see, e.g., [69], [70], [72]. The electron density, 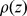, is approximated by a 1-dimensional Fourier analysis [73]: 
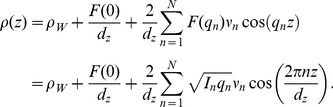
(1)



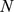
 is the highest order of the Bragg peaks observed in the experiment and 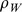 the electron density of bulk water. The integrated peak intensities, 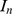, are multiplied by 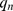 to receive the form factors, 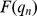 [102], [103]. The bilayer form factor 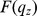, which is in general a complex quantity, is real-valued in the case of centro-symmetry. The phase problem of crystallography, therefore, simplifies to the sign problem 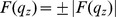 and the phases, 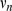, can only take the values 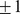. The phases 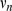 are needed to reconstruct the electron density profile from the scattering data following Equation (1). When the membrane form factor 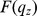 is measured at several 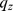 values, a continuous function, 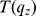, which is proportional to 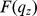, can be fitted to the data [102]–[105]: 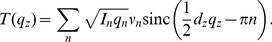
(2)


In order to determine the phases quantitatively, the form factor has to be measured at different 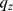-values using the so-called “swelling technique” [104] or by measuring the bilayer form factor at different scattering contrast conditions when using neutron diffraction [106], [107]. By fitting the experimental peak intensities and comparing them to the analytical expression for 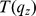 in Eq. (2), the phases, 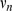, could be assessed. Good agreement was obtained, as shown in Figure 4.

To put 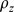 on an absolute scale, the electron densities for the pure lipid DMPC/DMPS bilayers and the DMPC/DMPS/cholesterol and DMPC/DMPS/melatonin samples were scaled to fulfil the condition 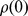 = 0.22 e/Å^3^ (the electron density of a CH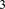 group) in the center of the bilayer. The electron density between two stacked bilayers, 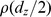, is often scaled to the electron density of bulk water, which is a good approximation in single component model membranes. The addition of DMPS to the DMPC bilayers, a phospholipid whose net 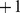 charge, may result in a nonuniform distribution of head groups at the head group-water interface. In addition, membrane embedded or surface bound peptides are likely to change the structure at the water/bilayer interface. The electron densities were, therefore, scaled such that the integral of the electron density across the bilayer, multiplied by the area per lipid yielded the required number of electrons.

The average electron density of an amino acid is calculated to be 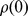 = 0.33 e/Å^3^. The electron densities at the bilayer center, 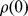, for the peptide samples were, therefore, tentatively scaled to values 0.22 e/Å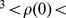 0.33 e/Å^3^ until a good agreement with the peptide structure calculations was achieved. Best agreement for the A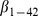 peptide in Figure 6 b) was obtained for a value of 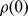 = 0.33 e/Å^3^, indicative that mainly amino acids reside in the bilayer center, in agreement with the proposed position. A value of 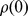 = 0.22 e/Å^3^ provided good agreement between experiment and calculation for the A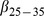 fragment in Figure 6 a), indicative that this peptide does not take a trans-membrane position.

The electron density is defined by 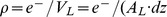), where 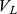 is the volume of a unit cell, 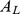 the area and 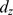 the lamellar spacing, i.e., the size of the unit cell. The integral 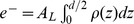 gives the total number of electrons in one leaflet. For the 97 mol% DMPC/3 mol% DMPS bilayers at 50% RH, this number is calculated to be 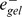 = 443.94, corresponding to the lipid molecules and 7 water molecules. This is in very good agreement with the results by Nováková, Giewekemeyer, and Salditt [108], who determined the number of tightly bound water molecules in some two-component lipid bilayers from X-ray reflectivity. 8 water molecules were determined for a 4∶1 DPPC/DPPS bilayer. 25 water molecules per lipid molecule were reported in fully hydrated fluid lipid bilayers [76], [108] and used for the electron density calculation in the 100% RH scans.

The 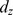-spacing between two neighboring membranes in the stack was determined from the distance between the well developed Bragg reflections (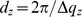) along the out-of-plane axis, 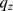, as shown in Figure 4, for example. The peaks were well fit by Gaussian peak profiles. Up to a peak order 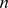 of 10 was observed in the out-of-plane data. Note that not all diffraction orders are necessarily observed for the different 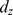-spacings as their scattering intensity depends on the form factor of the bilayers and oscillates between zero and maximum intensity as a function of 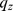.
